# Subcellular Localization and RNA Interference of an RNA Methyltransferase Gene from Silkworm, *Bombyx Mori*

**DOI:** 10.1155/2008/571023

**Published:** 2008-05-21

**Authors:** Zuoming Nie, Ruobing Zhou, Jian Chen, Dan Wang, Zhengbing Lv, Pingan He, Xuedong Wang, Hongdan Shen, Xiangfu Wu, Yaozhou Zhang

**Affiliations:** Institute of Biochemistry, College of Life Sciences, Zhejiang Sci-Tech University, Second Avenue, Xiasha, Hangzhou 310018, China

## Abstract

RNA methylation, which is a form of posttranscriptional modification, is catalyzed by S-adenosyl-L-methionone-dependent RNA methyltransterases (RNA MTases). We have identified a novel silkworm gene, *BmRNAMTase*, containing a 369-bp open reading frame that encodes a putative protein containing 122 amino acid residues and having a molecular weight of 13.88 kd. We expressed a recombinant His-tagged BmRNAMTase in *E. coli* BL21 (DE3), purified the fusion protein by metal-chelation affinity chromatography, and injected a New Zealand rabbit with the purified protein to generate anti-BmRNAMTase polyclonal antibodies. Immunohistochemistry revealed that BmRNAMTase is abundant in the cytoplasm of *Bm5* cells. In addition, using RNA interference to reduce the intracellular activity and content of BmRNAMTase, we determined that this cytoplasmic RNA methyltransferase may be involved in preventing cell death in the silkworm.

## 1. Introduction

S-adenosyl-L-methionone-dependent RNA methyltrans-ferases (RNA MTases) are enzymes that use S-adenosyl-L-methionone (AdoMet) as the methyl donor group when catalyzing the methylation of RNA substrates [1–3]. Methylation of RNAs may modulate RNA maturation, alter RNA structure stability, and affect translation rates [4]. Several studies have indicated that almost all MTases have similar basic structures, however, the substrates targeted by various Mtases differ because of small differences in regions of the enzymes that contain the substrate binding site [5, 6]. The catalytic domain of MTases is highly conserved, including the common structural core and the AdoMet binding site, which accommodates the catalytic reaction and releases the final catalyzed product. The catalytic domain also plays an important role in stabilizing the conformation of the enzyme [7].

Due to variations in the binding sites for AdoMet, the binding methyl groups are allocated to different substrates; accordingly, AdoMet-dependent MTases are divided into five families (Class I–Class V) [8]. *BmRNAMtase*, which was derived from a *Bombyx mori* pupae stage cDNA library constructed in our laboratory, belongs to Class IV, otherwise known as the SPOUT (SpoU-TrmD) family of RNA MTases [9, 10]. The SpoU and TrmD families were previously considered to be independent families, however, amino acid sequence analyses have shown that members of these families share three common sequence motifs and also contain a “deep trefoil knot” motif in the C-terminus. Sequence analyses also indicate that mature SpoU proteins can potentially be targeted for several types of posttranslational modifications including N-myristoylation, cAMP- and cGMP-dependent protein kinase phosphorylation, and glycosylation.

In this study, we performed bioinformatics analysis of BmRNAMTase and expressed the *BmRNAMTase* open reading frame in *E. coli* BL21 (DE3). Rabbits inoculated with the recombinant protein generated high titer polyclonal antibodies, which we used to determine the subcellular localization of BmRNAMTase in a *Bombyx mori* cell line by confocal immunofluorescence microscopy. Finally, we also investigated the effect of *BmRNAMTase* dsRNA on *Bm5* cell growth and determined that silencing BmRNAMTase activity resulted in increased cell death but did not induce apoptosis.

## 2. Materials and Methods

### 2.1. The Bioinformatic Analysis of BmRNAMTase

The similarity analysis of nucleotide and protein sequences was carried out using the BLASTN and BLASTP algorithms (NCBI). The deduced amino acid sequence and characteristics of BmRNAMTase were analyzed using the Expert Protein Analysis System (http://www.expasy.org/). The phylogenetic tree for BmRNAMTase and additional RNA methylases was generated using the MEGA 3.1 software.

### 2.2. Construction of Recombinant Plasmid

To amplify the open reading frame of *BmRNAMTase* from the cDNA fragment in a silkworm pupa cDNA library constructed by our laboratory [11], we performed the polymerase chain reaction (PCR) using upstream oligonucleotide primer P1 and downstream oligonucleotide primer P2 (Table 1). We digested the PCR product with *Sac*I and *Sal*I (Promega, Madison, USA) and ligated it into *Sac*I*- Sal*I-digested pET-28a(+) expression vector. The recombinant plasmid was transformed into *E. coli* BL21 (DE3).

### 2.3. Expression and Purification of Recombinant Protein

The transformed strain was grown at 37°C in Luria-Bertani medium containing kanamycin (100 *μ*g/*μ*l) until the optical density at 600 nm of the culture reached 0.6. Isopropyl-1-thio-*β*-D-galactopyranoside (IPTG) (Promega) was added to the culture to a final concentration of 1 mM, and cells were grown for an additional 5 hours before harvesting. The cells were pelleted by centrifugation; the cell pellet was suspended in phosphate-buffered saline (PBS), and the cells were lysed by sonication. The lysed cells were centrifuged and the supernatant was loaded onto an SP-Sepharose (G200) column; fractions collected from the Sepharose column that contained the recombinant protein were loaded onto an Ni^2+^-SephadexTM G-25 Superfine (HiTrapTM) desalting column (Amersham, Piscataway, USA) [12]. Purified BmRNAMTase was analyzed by SDS-PAGE.

### 2.4. Preparation of Polyclonal Antibodies and Western Blot Analysis

Rabbits inoculated with purified recombinant protein generated high titer anti-BmRNAMTase polyclonal antibodies, as determined by ELISA [12]. We determined the specificity of the polyclonal antibodies for recombinant BmRNAMTase and for BmRNAMTase in silkworm pupae by Western blot analysis [12].

### 2.5. Subcellular Localization of BmRNAMTase

*Bm5* cells were seeded into a dish for confocal microscopy. Cells were rinsed twice with 1mL PBS, fixed in 3.7% formaldehyde at 25°C for 25 minutes, and rinsed three additional times with PBS. The fixed cells were blocked in a 3% BSA solution at 25°C for 2 hours. The cells were then incubated with anti-BmRNAMTase serum (dilution, 1 : 50) at 4°C for 12 hours. After three 10-minute washes with PBST (PBS+0.05% Tween-20), the cells were incubated with Cy3-labelled goat antirabbit antibody (dilution, 1 : 2000) (Promega) at 37°C for 2 hours, and finally cells were incubated with 4′-6-Diamidino-2-phenylindole (DAPI) (dilution, 1 : 2000) (Promega) at room temperature for 10 minutes. Following two 10-minute washes with PBST and another two 10-minute washes with PBS, cells were observed under a Nikon ECLIPSE TE2000-E confocal microscope and images were analyzed using EZ-C1 software.

### 2.6. RNAi

To determine BmRNAMTase function, we transfected *Bm5* cells with a BmRNAMTase dsRNA. The BmRNAMTase ORF was amplified by PCR using primers containing T7 promoter sequences (P3 and P4) (Table 1). The primer used for the sense strand was P3 and for the antisense strand was P4. pET-28a(+)-BmRNAMTase was used as the template for the PCR, and we amplified the two single strands using two primer pairs: P3 and P2, and P4 and P1 (Table 1). Using the T7 RiboMAXExpress RNAi System kit (Promega), we synthesized the dsRNA and purified PCR products according to the manufacturer's instructions. The products were quantified spectrophotometrically at 260 nm and confirmed by agarose gel electrophoresis. Silkworm *Bm5* cells were transfected with the BmRNAMTase dsRNA using liposomes (Invitrogen, Carlsbad, USA), and cytoactivity was determined after treatment with different concentrations of dsRNA (1.2, 2.4, 3.6, 4.8, 6.0, 7.2, and 8.4 *μ*g/*μ*l) and the effectiveness of different durations of the RNAi treatment (12, 24, 36, 48, 60, 72, 84, and 96 hours) was determined using MTT [13]. Using a cell counter, we determined when the cell density reached about 1 × 10^5^ units/mL after the RNAi treatment. At that time, we extracted the total protein from the cells and determined the change in BmRNAMTase content in the cells caused by the RNAi treatement using ELISAs. The effect of the treatment on cell growth and death was determined by examining the chromosomal DNA; the appearance of a characteristic DNA ladder signifies cell apoptosis [13].

## 3. Results

### 3.1. Bioinformatic Analysis of BmRNAMTase Sequence

**Figure 1:**
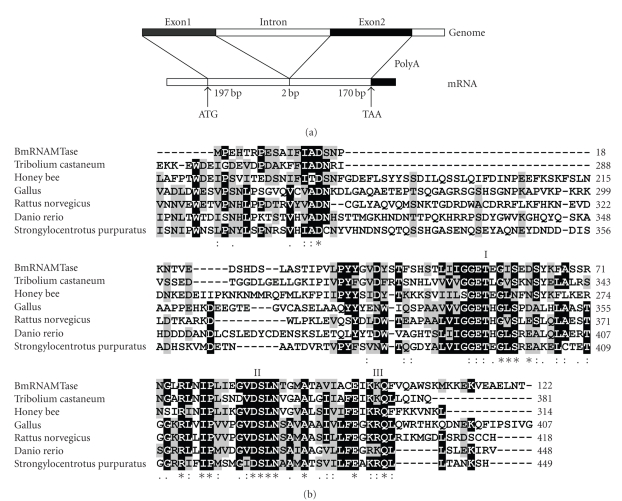
Bioinformatics analysis of the silkworm *BmRNAMTase* gene. (a) A schematic representation of the silkworm *BmRNAMTase* gene; this gene contains two exons. (b) Homologous alignment between deduced amino acid sequences of BmRNAMTase and proteins derived from other species. The asterisks indicate the identical amino acids; the black areas indicate the conserved domains of SpoU-methylase and SpoU. I, II, III represent conserved motifs, respectively.

We obtained a unique cDNA containing a 369 bp open reading frame that encodes an AdoMet-dependent RNA methyltransferase protein containing 122 amino acid and having a predicted molecular weight of 13.88 kd from a silkworm pupae cDNA library constructed in our laboratory (GenBank accession number DQ813500). This gene, *BmRNAMTase,* contains two exons and one intron (Figure 1(a)) and encodes an RNA methyltransferase that contains a domain that is highly conserved (SpoU) in similar genes in other species (Figure 1(b)).We have determined that *BmRNAMTase* is the first reported *Bombyx mori* gene to encode an RNA methyltransferase geneusing the BLAST software available on the NCBI web site.

**Figure 2:**
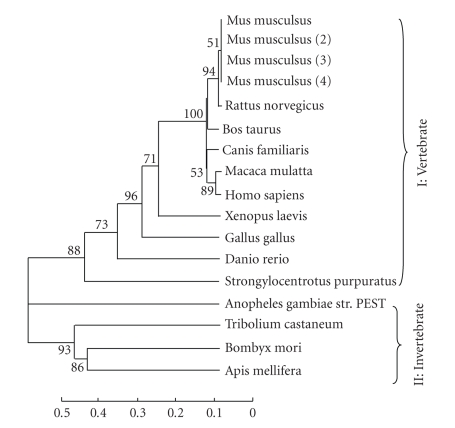
The phylogenetic tree for RNA MTases from various species. The node is the bootstrap value of 1000 repeats; scale represents the distance of descent.

The evolutionary relationships of a protein can provide important information about its functions. Therefore, we performed a phylogenetic tree analysis of RNA MTases to identify homologs of BmRNAMTase in other species (Figure 2). The phylogenetic tree shows the evolutionary relationships of the structural domains of RNA MTases from various species. It contains two primary branches: I and II. Branch I is primarily composed of vertebrate homologs, and branch II includes only invertebrate homologs. The vertebrate homologs are more highly evolutionary conserved than the invertebrate homologs. Therefore, we presumed that the functional domains in RNA MTases changed significantly during the evolutionary split between invertebrates and vertebrates. The species that are most closely related showed a high degree of homology in the structural domain within the RNA MTases.

### 3.2. Cloning, Expression, and Purification

**Figure 3:**
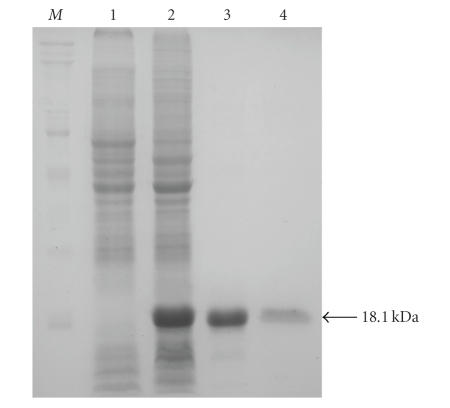
SDS-PAGE analysis of BmRNAMTase. *Lane* 1, the control of BL21 (pET-28a(+)) after induction with IPTG, *Lane* 2, BL21 (pET-28a(+)-BmRNAMTase) after induction with IPTG, *Lane* 3, pooled fractions from the SP-Sepharose(G200) column, *Lane* 4, pooled fractions from the His-Trap column; *Lane* M, protein markers (TaKaRa). Position of the 18.1 kd protein standard is indicated.

We cloned the *BmRNAMTase* ORF into the expression vector pET-28a (+). We then transformed *E. coli to* the recombinant plasmid, grew transformed cells in culture to the appropriate density, and used IPTG to induce expression of an 18.1 kd recombinant protein containing a His-tag (data not shown). We purified the recombinant protein using successive steps of SP-Sepharose (G200) column chromatography followed by metal-chelating affinity chromatography on a column containing Ni^2+^-SephadexTM G-25 Superfine. We obtained a yield of approximately 4.0 mg of purified protein from a one liter *E. coli* culture. The purity of the recombinant protein, as estimated by SDS-PAGE, was greater than 90% (Figure 3).

### 3.3. Immunohistochemistry Analysis

**Figure 4:**
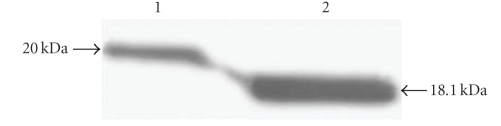
Western blot analysis of purified recombinant protein and protein derived from silkworm pupae. *Lane* 1, BmRNAMTase extracted from silkworm pupae; *Lane* 2, the purified recombinant protein.

ELISAs showed that the titer of the polyclonal antibody is about 1 : 51, 200, and Western blot analyses revealed that the antibody is highly specific for BmRNAMTase. We also determined that BmRNAMTase is highly expressed in silkworm pupae. However, Western blot analyses indicated an apparent molecular weight of about 20 kd, which is 6 kd greater than the predicted molecular weight (Figure 4). To address this inconsistency, we used http://au.expasy.org/prosite/ to identify potential sites in the BmRNAMTase amino acid sequence that might be posttranslationally modified. We found that BmRNAMTase contains two potential sites for Casein kinase II phosphorylation: 5–8 (TRPE) and 21–24 (TVED); three potential sites for N-myristoylation: 40–45 (GVDYST), 73–78 (GLRLNI), and 90–95 (GMATAV); and two potential sites for Protein kinase C phosphorylation: 64–66 (SYK) and 69–71 (SSR). The amino acid sequence of BmRNAMTase also contains several potential glycosylation sites, which is consistent with predictions made using http://www.cbs.dtu.dk/services/NetNGlyc/. It is possible that posttranslational modifications are responsible for the increased apparent molecular weight of BmRNAMTase.

### 3.4. BmRNAMTase is Abundant in the Cytoplasm of Bm5 Cell

**Figure 5:**
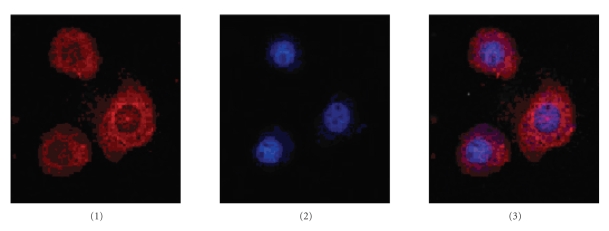
The subcellular localization of BmRNAMTase in *Bm5* cells. 1, immunostaining with anti-BmRNAMTase antibodies; 2, image of nuclei using the nucleic acid stain DAPI; 3, overlay of images 1 and 2.

To determine the subcellular localization of BmRNAMTase, we performed immonohistochemistry, using the anti-BmRNAMTase polyclonal antibodies as the primary antibody and Cy3-conjugated goat antirabbit antibodies as the secondary antibody, to locate BmRNAMTase in *Bm5* cells. We observed immunostained cells under a Nikon ECLIPSE TE2000-E confocal microscope and determined that BmRNAMTase is found mostly in the cytoplasm; it is particularly enriched in the inboard of karyotheca, and little BmRNAMTase is found in the karyon (Figure 5). The localization of BmRNAMTase suggests that it may play a role in posttranscriptional modification of RNAs.

### 3.5. Effect of RNAi on Cell Growth

**Figure 6:**
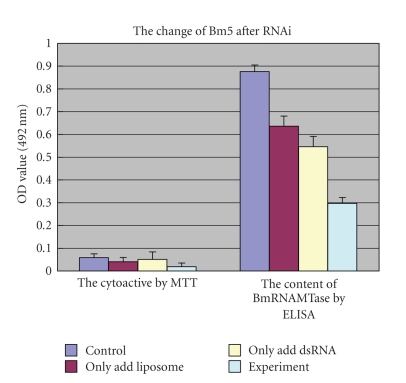
Cytoactivity and BmRNAMTase content in *Bm5* cells after RNAi treatment.

**Figure 7:**
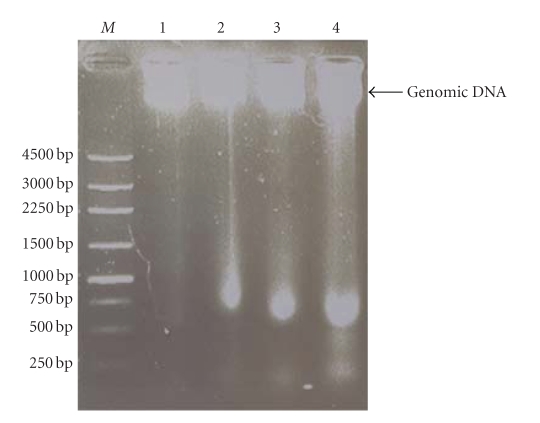
Determination of the apoptotic morphology of the cell with DNA ladder. M, 250 bp DNA marker; *Lane* 1, control; *Lane* 2, sample interfered with only lipid; *Lane* 3, sample interfered with only dsRNA; *Lane* 4, sample interfered with lipid and dsRNA.

MTT experiments demonstrated that the optimal concentration of the *BmRNAMTase* dsRNA for use in RNAi experiments is 6.0 *μ*g/*μ*l and that the optimal duration of the interference is 72 hours (data not shown). We determined cytoactivity by MTT and determined BmRNAMTase content in the cell by ELISA after cells had been treated for 72 hours (Figure 6). We also determined whether the treated *Bm5* cells had become apoptotic by examining chromosomal DNA for the appearance of a characteristic DNA ladder (Figure 7).

We concluded that the activity and content of BmRNAMTase in the *Bm5* cells decreased significantly following the RNAi treatment and that silencing the BmRNAMTase gene resulted in increased cell death (Figure 6). However, we did not observe the formation of a DNA ladder in the chromosomal DNA, which is characteristic indicator of apoptotic cells [13]. The appearance of the chromosomal DNA in the RNAi-treated cells indicated that cell death did increase during the treatment, however, it also demonstrated that there was no significant increase in apoptotic cell death in the treated cells. The changes in the activity and content of BmRNAMTase in the cells were possibly due to necrotic cell death.

## 4. Discussion

In the present study, we discovered the first *BmRNAMTase* gene in a silkworm pupae cDNA library. We expressed a recombinant fusion BmRNAMTase protein in the *E. coli* BL21 (DE3) strain, purified the recombinant protein, and used it to generate anti-BmRNAMTase polyconal antibodies for use in determining the subcellular localization of BmRNAMTase. Immunohistochemistry indicated that the majority of BmRNAMTase is found in the cytoplasm of *Bm5* cells. In addition, RNAi experiments indicated that silencing *BmRNAMTase* results in increased cell death.

RNA MTases collectively form an enzyme superfamily [14, 15] that has important biological functions. Methylation is a common post-transcriptional modification of RNAs; this modification can mediate control of translational processing. Prerequisites for RNA methylation are the availability of AdoMet-dependent MTases, which contain a common catalytic core and binding sites for the substrate, and both AdoMet and the substrate that corresponds to the binding site in the enzyme; binding sites vary slightly, depending on the substrate. Several studies on the mechanism of AdoMet-dependent MTase recognition of the substrate have been completed [16]. Recently, studies have focused on identifying catalytic and functional domains as well as determining the specific RNA sequences recognized in targeted substrates [17, 18]. The catalytic characteristics of several RNA AdoMet-dependent MTases have been investigated extensively, however, the mechanism responsible for recognition of substrates remains obscure.

In this study, we determined that inhibiting BmRNAMTase activity by depleting its content through RNA interference results in increased cell death (Figures 6 and 7). Analysis of the amino acid sequence revealed that the functions of BmRNAMTase might be similar to those of other members of the SpoU protein family. The key amino acid residues in SpoU family proteins are: R41, S150, P143, E124, N35, and S37 [7]. By comparing the BmRNAMTase sequence to those of other RNA MTases using CLUSTALW we determined that the key amino acid residues in BmRNAMTase may be R75, S61, P79, E91, N88, and S86 and that a possible function for BmRNAMTase is catalyzing the methylation of the 2′-OH at guanosine 18 in tRNAs.

**Figure 8:**
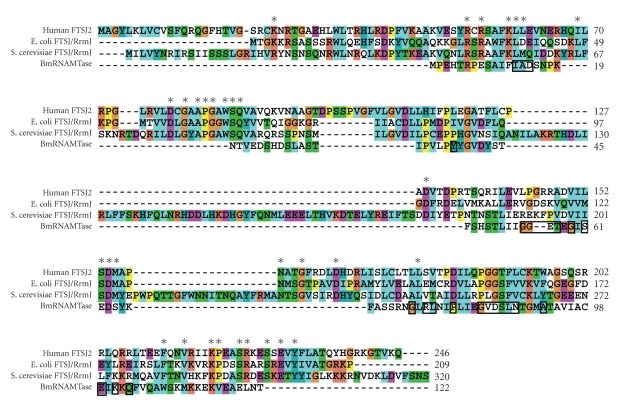
Alignment of BmRNAMTase and another class of RNA methyltransferases. Asterisks show the conserved sequences of ribosomal RNA methyltransferases; the conserved sequences of SpoU family proteins are boxed in BmRNAMTase sequences.

Subcellular localization of BmRNAMTase in *Bombyx mori* by confocal immunofluorescence microscopy indicated that the protein is concentrated in the cytoplasm (Figure 5), which differs significantly from the observed subcellular distribution of FTSJ2, a novel human RNA methyltransferase [19]. FTSJ2 shares significant sequence homology with FtsJ/RrmJ, an *E. coli* 23S rRNA uridine-2′-O-methyltransferase [20] and is primarily found in the nucleolus. BmRNAMTase shares significant sequence homology with SpoU family proteins, which primarily catalyze the transfer of a methyl group from AdoMet to the 2′-OH of guanosine 18 in tRNAs [21]. However, BmRNAMTase and other classes of RNA methyltransferases show different conserved sequences (Figure 8). The existence of differing conserved sequences in various classes of methylases is probably necessary to form different functional domains and binding sites for targeting and modifying different substrates. Alternative conserved amino acid sequences might also contribute to the differences in subcellular localization observed for various methylases.

We have studied the characteristics of BmRNAMTase and established a foundation for further functional studies on this enzyme. Western blot analyses have revealed an apparent molecular weight for BmRNAMTase in a silkworm pupa that is 6 kd greater than the predicted molecular weight; determining the underlying reason for this discrepancy requires further investigation. In addition, more study is necessary to determine the catalytic domain and the mechanism of substrate recognition of BmRNAMTase.

## Figures and Tables

**Table 1:** The primers used in the experiments.

Name	Sequences	The instead of underline	Function
P1	5′-GGAGCTCATGCCTGAACATCAAG-3	*Sac*I	Amplifying the fragment of *BmRNAMTase*
P2	5′-GGTCGACTTATGTGTTAAGTTCTG-3′	*Sal*I	
P3	5′-GGATCCTAATACGACTCACTATAGA	T7 promoter	Synthesizing the dsRNA of *BmRNAMTase*
	TGCCTGAACATACAAGA-3′		
P4	5′-GGATCCTAATACGACTCACTATAGT	T7 promoter	
	TATGTGTTAAGTTCTGC-3′		
